# Continuous positive airway pressure therapy for obstructive sleep apnoea and psychotropic drug use: a retrospective observational matched-cohort study

**DOI:** 10.1038/s41598-018-32142-6

**Published:** 2018-09-20

**Authors:** Angélique Delbarre, Frédéric Gagnadoux, Bénédicte Gohier, Nathalie Pelletier-Fleury

**Affiliations:** 1 Center for research in Epidemiology and Population Health (CESP), Université Paris-Saclay, Université Paris-Sud, UVSQ, Villejuif, France; 2 Université Bretagne Loire, CHU d’Angers, Département de Pneumologie, Angers, France; 3 Inserm UMR, 1063 SOPAM Angers, France; 4 Université Bretagne Loire, Laboratoire de Psychologie des Pays de Loire EA, 4638 Angers, France; 5 CHU d’Angers, Service de Psychiatrie et Addictologie, Angers, France

## Abstract

Patients with obstructive sleep apnoea (OSA) frequently present symptoms of depression, anxiety and insomnia and continuous positive airway pressure (CPAP) can improve these symptoms. Using a real-world administrative database, we analysed the trend of psychotropic drug use (antidepressants, anxiolytics and hypnotics) on a long-term period in OSA individuals before-after CPAP initiation. A total of 869 OSA individuals to whom psychotropic drugs were prescribed were followed for 4 years. They were matched to 2,607 non-OSA individuals, who were similar in terms of demographics, chronic diseases and care consumption. Generalized estimating equations models were used to compare psychotropic drug defined daily doses (DDD). Results showed no significant differences in mean trends of psychotropic drug DDD between OSA individuals and non-OSA matched controls during the three years following CPAP initiation. Only time had a significant effect on DDD, which decreased in both groups: −9% in Y1 and −17% in Y3, compared to Y0 (p-values < 0.0001). Hence, CPAP therapy does not result in an earlier decrease of psychotropic drug use in OSA individuals compared to non-OSA matched controls. Further studies are needed to analyse long-term psychotropic drug use, particularly in non-adherent OSA individuals.

## Introduction

Obstructive sleep apnoea (OSA) is a highly prevalent disease characterized by recurrent episodes of partial or complete upper airway obstruction during sleep, leading to intermittent hypoxia and sleep fragmentation. The reported prevalence of OSA has increased over time, and it is partly due to the rising rates of obesity^1^. Most recent estimates suggest that 13% of men and 6% of women experience clinically significant OSA^2,3^. Its diagnostic requires symptoms (e.g. daytime sleepiness, snoring, witnessed apnoeas) or associated medical or psychiatric disorder coupled with at least ≥5 respiratory events per hour of sleep during nightly recordings (polysomnography or respiratory polygraphy). Alternatively, a frequency of respiratory events ≥15 per hour satisfies the criteria, even in the absence of associated symptoms or disorders^4^. Nasal continuous positive airway pressure (CPAP) during sleep is recommended as first-line therapy for moderate-to-severe OSA^5^. It improves daytime alertness and health-related quality of life and lowers blood pressure^6–8^. There is a growing awareness that individuals with OSA frequently present symptoms of depression, anxiety and insomnia^9,10^. A study in 5 European countries estimated that approximately 17% of OSA patients have a diagnosis of major depressive disorder^11^ compared to 7% in the general population^12^. However, there is no consensus about the association between OSA and depression. It may constitute a causal relationship^13^ or may be due to overlapping diagnostic criteria (fatigue, loss of interest, decreased libido, and poor concentration)^14^. Regarding insomnia, evidence from clinical and research samples consistently suggests high rates of comorbidity between the two disorders^15,16^.

Limited evidence is available on the effect of CPAP on these symptoms^9,17–19^. Several studies have shown that CPAP therapy can induce an improvement of depressive symptoms^17–19^ while other studies have shown that CPAP had a moderate clinical effect on symptoms of depression and anxiety, but it was not significantly higher than that of the sham device^9^. Insomnia symptoms can also be improved by CPAP, as shown by Nguyên *et al*.^16^. However, none of these studies questioned if CPAP therapy could result in an earlier decrease of psychotropic drugs use. In this study, we examined the pattern of psychotropic drug use of OSA individuals under CPAP and compared this pattern with a non OSA population of psychotropic drug users. To address this issue, we used a real world administrative database to analyse the quantity and trends of psychotropic drug use (antidepressants, anxiolytics and hypnotics) on a long-term period in OSA individuals before-after CPAP initiation and to compare it to that of non-OSA matched individuals.

## Materials and Methods

### Databases

Two French national health databases, which are linked using a common anonymous beneficiary identifier, were used in the study: the *Échantillon Généraliste des Bénéficiaires* (EGB, the representative sample of the national population of health insurance beneficiaries) and the *Programme de Médicalisation des Systèmes d’Information en Médecine*, *Chirurgie et Obstétrique* (PMSI-MCO, the health administrative hospital discharge database for medical, surgical and obstetrics wards). The EGB is a representative sample consisting of 1/97^th^ of the health administrative database, which provides comprehensive information on all reimbursed ambulatory care (outpatient procedures, physician visits and drug dispensing claims) of the 67 million beneficiaries of the main French health insurance fund. It also includes patient demographic data and declared chronic diseases eligible for 100% health insurance coverage (OSA is not on this list). The PMSI-MCO is a health administrative hospital discharge database for medical, surgical and obstetrics wards providing comprehensive information on hospital care, including diagnoses (coded using the International Classification of Diseases, 10th revision), surgical and medical procedures. Codes from the classifications of medical procedures and of medical devices were used to identify individuals with polygraphies or polysomnographies and individuals treated by CPAP or mandibular advancement device (MAD), respectively (Supplementary Method S1).

Access to these databases is subject to prior training and authorization and was approved by the French independent data protection administrative authority (*Commission Nationale Informatique et Libertés*, *CNIL*, authorization No. 01-054). Written informed consent was not required, as all data were made anonymous.

### Study population

#### Individuals with OSA (treatment group)

Individuals were defined as having OSA if they had at least one reimbursement for CPAP and/or MAD. In France, CPAP therapy can be reimbursed by health insurance if the patient has an apnoea-hypopnea index (AHI) greater than 30 or between 15 and 30 associated with at least 10 arousals per hour of sleep or with a cardiovascular disease^20^.

All OSA individuals to whom psychotropic drugs were reimbursed during the year prior to CPAP initiation between 1^st^ January 2006, and 31^st^ December 2012 were selected. An algorithm developed by the National Health Insurance Fund and experts in the field was applied to identify individuals who used psychotropic drugs^21^, i.e., individuals treated with antidepressants, anxiolytics and/or hypnotics, without mental disabilities or psychiatric disorders (psychotic, mood neurotic, addictive, or other disorders) (see Supplementary Table S1).

They were followed for one year before CPAP and between one to three years after, depending on whether they were adherent to CPAP. Patients are considered adherent to CPAP if they use their CPAP device at least 3 hours per night; this is the necessary condition for them to be reimbursed by health insurance^22^. A meter reading of CPAP is regularly done depending on the frequency of the technicians’ home visits (every 3 to 6 months), and a mean value is computed.

Individuals were excluded from the treatment group if they had been reimbursed for a MAD at least once during the study period or if they had used CPAP for less than one year.

#### Control subjects

The control group consisted of non-OSA control subjects, not treated with CPAP or MAD, to whom psychotropic drugs were reimbursed between January 1, 2006, and December 31, 2012, without sleep recordings during the study period. For each year of inclusion (from 2006 to 2012), three control subjects were selected for each patient, matched for different types of characteristics collected the year of inclusion: demographics (age, gender), chronic diseases (diabetes, severe hypertension, stroke) and health care consumption (duration of psychotropic drug treatment, types of psychotropic drugs, number of physicians visits and number of hospitalizations).

#### Follow-up

**Figure 1:**
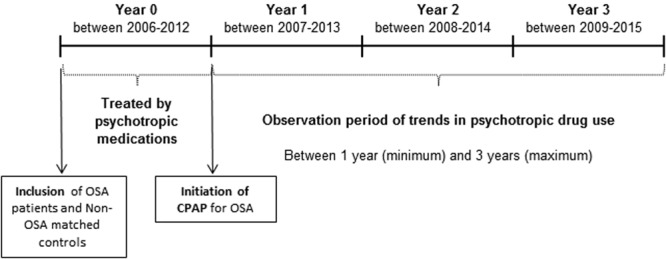
Population follow-up diagram. OSA: obstructive sleep apnoea, CPAP: continuous positive airway pressure.

A 4-year follow-up period was analysed, corresponding to 1 year prior to CPAP, labelled as Y0, and 3 years after, years 1, 2 and 3, labelled as Y1, Y2 and Y3, respectively (Fig. 1). The same year label was applied to the control group. For each year of follow-up and each individual, the quantity of psychotropic drugs was expressed as the number of Defined Daily Doses (DDD)^23^, which is assumed as the average maintenance dose per day for a drug used for its main indication in adults (see Supplementary Table S2, for an example). The types of end of follow-up were CPAP discontinuation (for OSA individuals), death, lost to follow-up (i.e., drop-out from the National Health Insurance scheme) and end of the study period.

### Data analysis

#### Matching of OSA individuals and non-OSA control subjects

The following variables were used for matching: year of inclusion (Y0), age, gender, types of drugs (antidepressants, anxiolytics and hypnotics) and the duration of psychotropic drug treatment (one year or longer). In addition, information on Y0 concerning the number of physician visits (general practitioner (GP), psychiatrist), number of hospitalizations (excluding hospitalizations for sleep recordings) and chronic diseases (diabetes, severe hypertension, stroke) was also collected^21^. Diabetes and severe hypertension were used as surrogates for obesity^24,25^.

Based on the demographics and medical variables listed above, which were collected the year before the initiation of CPAP (Y0), two matching methods were combined to select the matched control group from the non OSA population: the exact matching and the propensity score methods^26^. Matching methods minimize bias due to covariates by ensuring similar distributions in the treatment and control groups^27^. First, exact matching was used for Y0, age, gender, types of drugs and duration of psychotropic drug treatment. This method gives to each OSA patient, a potential group of non OSA controls that is identical to him/her on these covariates. Second, three controls from this latter group were selected for each OSA patient using the propensity score method. This method, which facilitates the matching by taking a large number of covariates into account, estimated the probability of having OSA and receiving CPAP therapy, i.e. to be in the patient group, for all patients and potential controls. For each Y0 (from 2006 to 2012), a logistic regression was computed using the following variables: age, gender, types of drugs (antidepressants, anxiolytics and hypnotics), duration of psychotropic drug treatment (1 year or longer), chronic diseases (diabetes, severe hypertension, and stroke), annual visits to doctors (GP, psychiatrist) and number of hospitalizations in the year. The matched controls had to be the patient’s nearest neighbours, defined by the smallest absolute differences between patient’s and control’s propensity scores. All controls constituted the non-OSA matched control group. The main characteristics of the study population were described with discrete variables, which were expressed as proportions and continuous variables that were expressed as the mean ( ± standard deviation (SD)).

#### Outcome of interest

The outcome of interest was the mean number of psychotropic drug DDD per individual (all types, antidepressants, anxiolytics, and hypnotics) for each year (Y0, Y1, Y2 and Y3). Generalized Estimating Equations (GEE) models were used to compare mean quantities and mean trends between OSA individuals and non-OSA matched controls, while considering the initial difference between groups and the effect of time. The longitudinal correlation is taken into account with a working correlation matrix that we have defined as autoregressive^28^.

All statistical analyses were performed with SAS Version 7.13 software (SAS Institute Inc. Cary, NC, USA) and R Version 3.4.2 (The R Foundation for Statistical Computing).

## Results

### Population and matching

**Figure 2:**
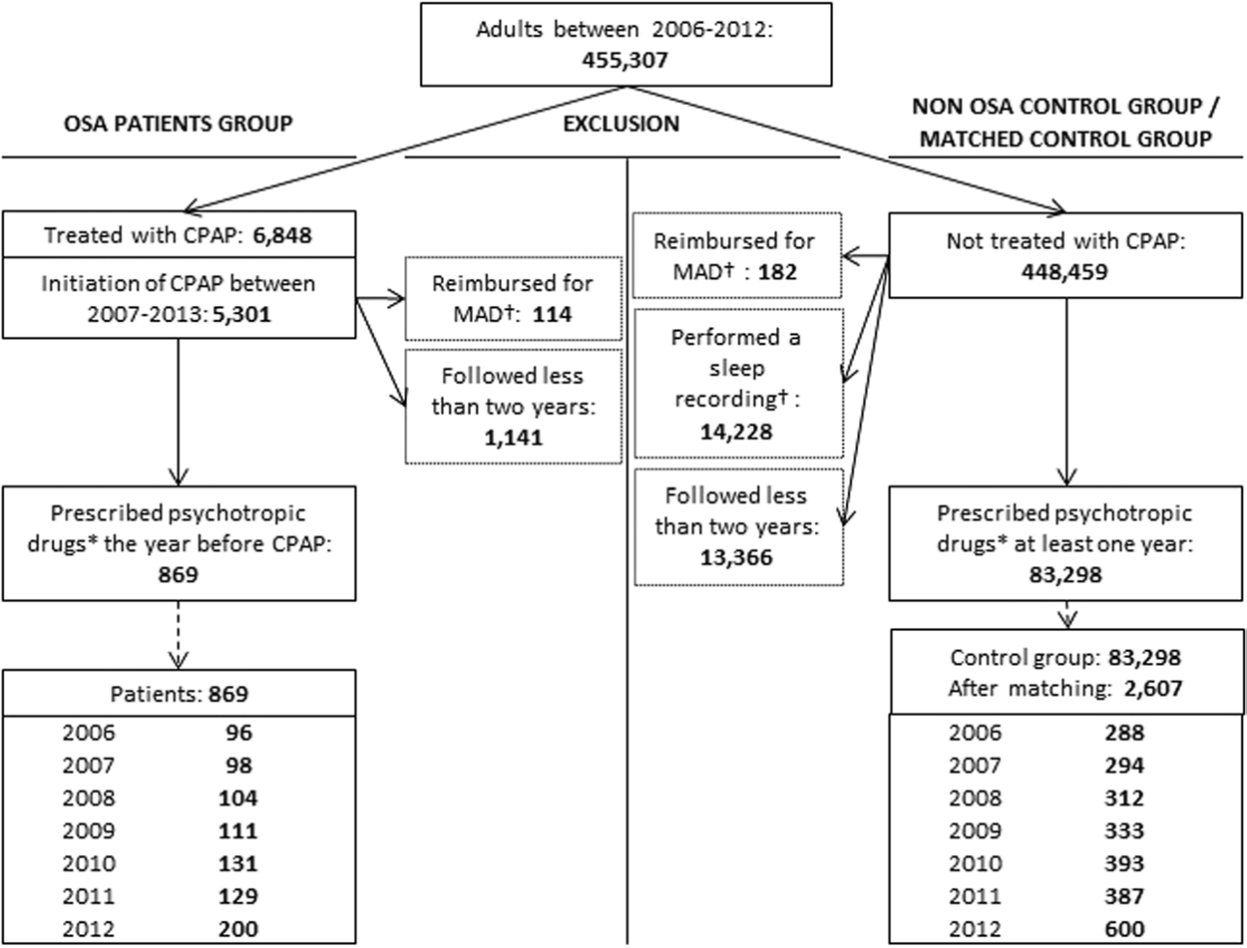
Study population flowchart. OSA: obstructive sleep apnoea, CPAP: continuous positive airway pressure, MAD: mandibular advancement device. †Reimbursement for MAD or sleep recordings were extracted from 2006 to 2015. *Antidepressants, anxiolytics and/or hypnotics in the absence of psychiatric disorders or neuroleptic prescription.

The study population consisted of 869 OSA individuals and 2,607 non-OSA matched controls (Fig. 2). Table 1 summarizes the baseline characteristics of the study population: one-half of OSA individuals were male, and they had a mean age of 60.4 ( ± 12.2) years. Antidepressants were the most commonly prescribed (62%) psychotropic drugs, followed by anxiolytics (45%) and hypnotics (29%). Comorbidities consisted of diabetes in almost one quarter of individuals, severe hypertension in 11%, and stroke in 3% of individuals. They visited the GP an average once a month and almost 16% of them discontinued CPAP therapy during the study period but after at least one year of treatment. As a result of exact matching, gender, age, types of psychotropic drug and duration of psychotropic drug treatment the year of inclusion (Y0) were identical between groups. The three controls for each OSA individual were reimbursed for the same types of psychotropic drugs at Y0, but the individual quantities of psychotropic drugs (number of DDD) might differ between them.

**Table 1:** Baseline characteristics of OSA individuals and non-OSA matched controls.

Characteristics	OSA individuals (n = 869)	Matched controls (n = 2,607)
**Gender, %**		
Male	53.62	53.62
Female	46.38	46.38
Age, years		
mean ( ± SD)	60.40 ( ± 12.24)	60.28 ( ± 12.76)
median [min; max]	60 [25; 89]	60 [20; 89]
**Types of psychotropic drugs, %**		
Antidepressants	62.14	62.14
Anxiolytics	45.11	45.11
Hypnotics	29.11	29.11
**Duration of psychotropic drug treatment, %**		
1 year	23.01	23.01
≥2 years	76.99	76.99
Comorbidities, %		
Diabetes	23.59	23.63
Severe hypertension	11.62	10.78
Stroke	3.34	2.99
**Annual number of physician visits, mean (SD)**		
Visits to the general practitioner	10.01 (6.59)	9.67 (6.55)
Visits to the psychiatrist	0.68 (3.53)	0.61 (4.38)
**Annual number of hospitalizations** ^**†**^		
mean ( ± SD)	1.09 (5.31)	0.98 (4.38)
**Type of end of follow-up, %**		
CPAP discontinuation after one year	15.88	NA
Death	2.30	5.26
Lost to follow-up	0.35	0.54
End of study period	81.47	94.21

OSA: obstructive sleep apnoea, CPAP: continuous positive airway pressure, SD: standard deviation. ^†^Excluding sleep recordings.

### Results of GEE models

The results of the GEE models, which modelled the annual mean DDD per individual, are presented in Table 2. The between-group difference at Y0 was expressed by the “group” covariate, the time effect was expressed by the “year” covariate and the effect of group after CPAP initiation was expressed by the “group × year” interaction. This interaction measured the effect of being in the OSA individuals group compared to the non OSA matched controls group at Y1 (initiation of CPAP), Y2 and Y3.

**Table 2:** Results of GEE models on OSA individuals and non-OSA matched controls.

Variable	Model 1: All psychotropic drugs		Model 2: Antidepressants		Model 3: Anxiolytics		Model 4: Hypnotics	
	Effect^†^ (p)	95% CI	Effect^†^ (p)	95% CI	Effect^†^ (p)	95% CI	Effect^†^ (p)	95% CI
**Group**								
Matched control	ref.	—	ref.	—	ref.	—	ref.	—
OSA	+ 4% (NS)	[−2%, + 11%]	+ 19% (***)	[ + 12%, + 27%]	−14% (***)	[−21%,−5%]	−9% (**)	[−17%,−1%]
**Year**								
0	ref.	—	ref.	—	ref.	—	ref.	—
1	−9% (***)	[−11%,−7%]	−8% (***)	[−11%,−5%]	−8% (***)	[−11%,−5%]	−11% (***)	[−14%,−7%]
2	−14% (***)	[−16%,−11%]	−14% (***)	[−17%,−10%]	−15% (***)	[−18%,−11%]	−12% (***)	[−16%,−8%]
3	−17% (***)	[−20%,−15%]	−16% (***)	[−20%,−12%]	−21% (***)	[−24%,−17%]	−16% (***)	[−20%,−11%]
**Group** × **Year**								
OSA × 1	−1% (NS)	[−6%, + 4%]	0% (***)	[−6%, + 6%]	−4% (NS)	[−11%, + 4%]	−1% (NS)	[−10%, + 9%]
OSA × 2	+ 1% (NS)	[−5%, + 7%]	+ 4% (***)	[−4%, + 13%]	−4% (NS)	[−13%, + 6%]	−5% (NS)	[−14%, + 6%]
OSA × 3	+ 1% (NS)	[−5%, + 8%]	+ 3% (***)	[−6%, + 12%]	+ 4% (NS)	[−7%, + 15%]	−8% (NS)	[−19%, + 4%]

GEE: generalized estimating equations, CI: confidence interval, p: P-value, OSA: obstructive sleep apnoea. NS: not significant, *p-value < 10%, **p-value < 5%, ***p-value < 1%. A logarithm function was used as link function. The estimated intercepts are not shown in the table. ^†^The multiplicative effect was computed from a transformation of the estimated coefficients β: Effect = (1 - β) × 100.

**Figure 3:**
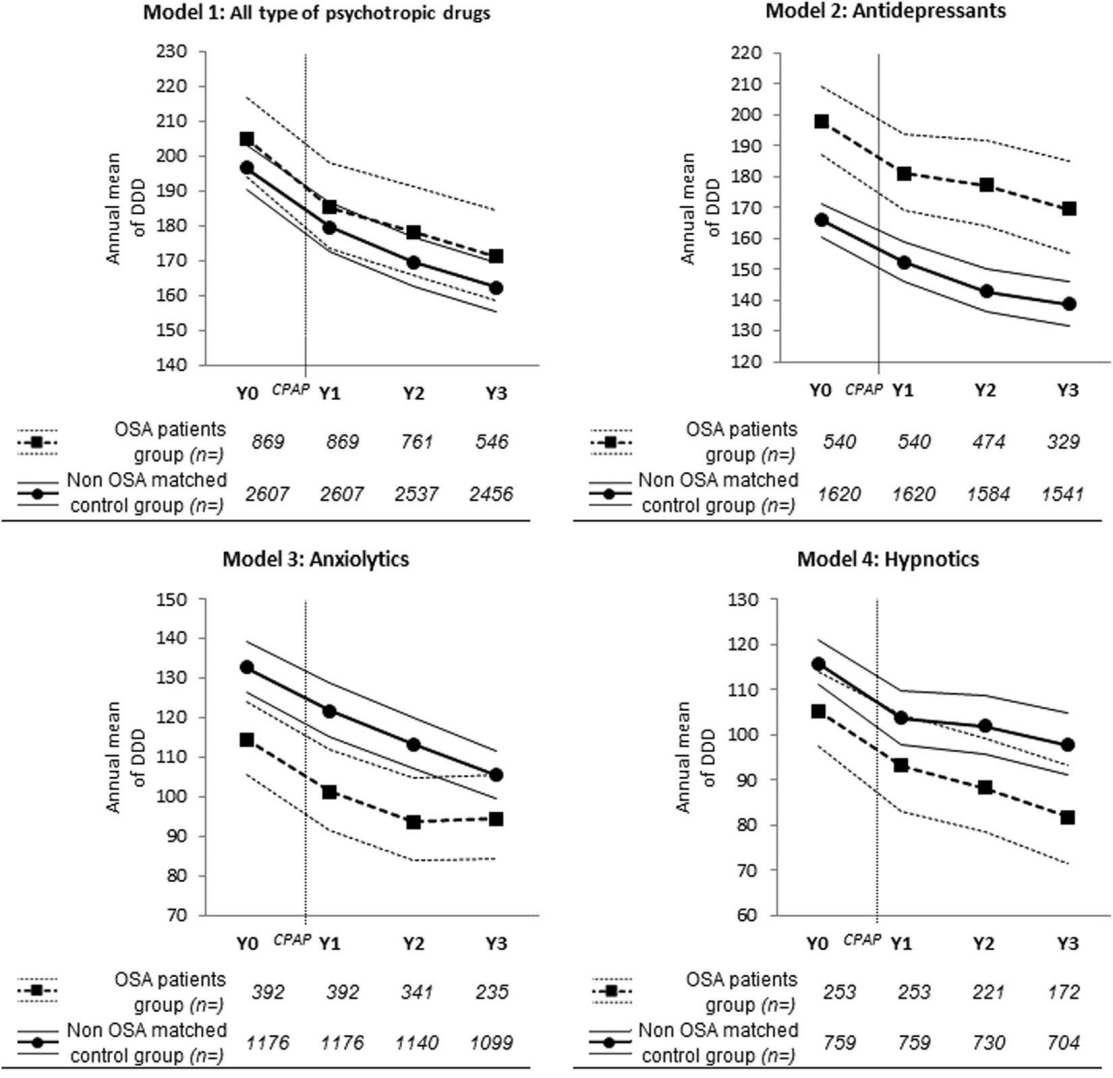
Results of GEE models on OSA individuals and non-OSA matched controls: estimated annual mean psychotropic drug DDD per group and their 95% CI. GEE: generalized estimating equations, DDD: defined daily doses, CI: confidence interval, OSA: obstructive sleep apnoea. Mean and 95% confidence intervals were computed with Generalized Estimating Equations (GEE) models.

At baseline, i.e., the year before initiation of CPAP, no significant difference of annual mean of DDD of all types of psychotropic drugs (model 1) per individual was observed between groups at inclusion. The differences between groups at baseline were significant for models 2, 3 and 4 (antidepressants, anxiolytics and hypnotics, respectively) (p-values < 0.0001). Concerning the use after initiation of CPAP in model 1, no significant interaction “group × year” was observed. This finding indicates that there were no significant differences in mean trends of psychotropic drug use between OSA individuals and non-OSA matched controls during the three years following CPAP initiation (Y1, Y2 and Y3). Only time had a significant effect on psychotropic drug use, which decreased in both groups: −9% in Y1 and −17% in Y3, compared to Y0 (p-values < 0.0001). In models 2, 3 and 4, the results were similar: the time effects were significant (p-values < 0.0001), but the interactions were not significant. Fig. 3 presents a visual representation of the 4 GEE models and the estimated annual mean psychotropic drug DDD per individual, together with their 95% CI, in the OSA individuals group and the non OSA matched controls group from Y0 to Y3.

## Discussion

To the best of our knowledge, this is the first study to evaluate the trends of psychotropic drug use before and after CPAP therapy in OSA individuals. The analysis was performed over a long-term period, based on a real-world database using anatomical therapeutic chemical (ATC) codes to identify the drugs and an internationally recognized method, the DDD, to quantify them. Moreover, the analysis used exact and propensity score matching methods, which represent the most appropriate methods in observational studies to control for confounding factors when estimating treatment effects^29,30^. The main finding of the study was that during the years following CPAP therapy, the trend of psychotropic drug use was similar to that of non-OSA matched controls on the same period: −17% (95% CI [−20%,−15%]) after three years of use compared to the year before initiation. It would have been interesting to also analyse the evolution of associated depressive, anxiety and/or insomniac symptoms of the study population. Unfortunately this was not feasible because the medico-administrative database we used did not contain any information on clinical symptoms. Furthermore many papers have already studied this issue^9,16,19^. However these papers did not question the evolution of psychotropic drug use after CPAP initiation. Only two studies were found in the literature that mentioned cessation of treatment under CPAP^17,18^. The study by Edwards *et al*. examined depressive symptoms before and after 3 months of CPAP^17^. The authors showed that depressive symptoms decreased, but one-half of the patients who were previously prescribed antidepressants remained under treatment over the course of the study. In the study by Gagnadoux *et al*., after an average of 529 days of CPAP therapy, 46% of OSA patients with depressive symptoms and who received antidepressants therapy at baseline (n = 89) were taken off the drugs during the follow-up period^18^. However, these studies were not specifically designed to detail psychotropic drugs evolution and were based on declarative data. Several hypotheses can be advanced to explain our findings. First, the prescribers’ attitudes could affect the results, as it is well established that it is easier to set up a treatment than to re-evaluate the symptoms in order to decrease it^31^. However, there is no reason to believe that the prescribers’ attitudes would be more common in patients with OSA. We could even think the opposite in our study as GP consultations were slightly more frequent in OSA individuals, providing doctors with the opportunity to re-evaluate the treatments (see Supplementary Table S3). Another explanation might be compliance with CPAP, which may be improved by antidepressants^13^ or non-benzodiazepine hypnotics without worsening AHI^32,33^. Finally, the persistence of symptoms related to OSA-independent depressive disorders or OSA-related comorbidities, such as obesity, metabolic syndrome^34^, or cardiovascular diseases^35,36^, might be the most likely explanation.

This study has several limitations that need to be considered when interpreting these findings. First, using the algorithm of health insurance, the study population was composed of individuals under psychotropic drugs, but without any observed psychiatric disorder^21^. However, it cannot be ruled out that individuals with undiagnosed (or non-coded) psychiatric disorders are included in the study. If this is the case, this potential bias would be similar in both groups and would not impact the study results. Second, the use of data obtained from an administrative claims database is associated with the difficulty of assessing adherence. We believe that this limitation was overcome in the present study, as reimbursement of CPAP is dependent on adequate adherence in France, i.e. that OSA individuals who are reimbursed used CPAP at least 3 hours per night^22^. However, we did not know if there was continued adherence during the follow-up period, as it is the case in most studies dealing with adherence to CPAP therapy found in the clinical literature^16,18^. This variation in adherence over time if any may have led to underestimate the effect of CPAP on psychotropic drug use. Third, exact and propensity score matching methods were used because they facilitate the construction of two balanced groups^29,30^. Unfortunately, some confounding factors that could potentially influence the results of matching were not available, for instance, OSA severity, obesity or lifestyle factors (marital status, socioeconomic status). Diabetes and hypertension were used as surrogates for obesity, as they are highly correlated with body mass index^24,25^ which was not available in our data. Finally, we cannot exclude that CPAP is effective on psychotropic drug use by preventing an increase in their use. Indeed, a good adherence to CPAP would remove the symptoms of ongoing OSA and make the OSA group more similar to the matched non-OSA controls. This could explain the similar evolutions between groups. This potential effect of CPAP cannot be observed without comparing treated and untreated OSA individuals. However, from an ethical perspective, this may be difficult to do. A comparison of psychotropic drug users under CPAP with psychotropic drug users who have stopped CPAP has been performed. Unfortunately, even if the trends of psychotropic drug use of these OSA individuals looked similar to that of OSA patients adherent to CPAP therapy, we could not conclude as this latter group was too small (n = 151) to carry out correct matching and perform robust analyses (see Supplementary Figure S1).

## Conclusions

CPAP therapy does not result in an earlier decrease of psychotropic drug use in OSA individuals after a 3-year follow-up. Further studies are needed to analyse long-term psychotropic drug use in non-adherent OSA patients.

## Electronic supplementary material

Supplementary Information

## Data Availability

Access to these medico-administrative databases is under control of the National Institute of Health Data (Institut National des Données de Santé, http://www.indsante.fr/), and approved by the Independent Data Protection Administrative Authority (Commission Nationale Informatique et Libertés, CNIL, authorization No. 01-054). All researchers from non-profit and profit organizations may request access to the data and ask for training to carry out studies with public interest. The protocol assessment of these studies is shorter for non-profit organizations (public research institutes as the National Institute of Health and Medical Research (Institut National de la Santé et de la Recherche Médicale, Inserm), French ministry of health, regional public health authorities). Information and request for access to the data set may be sent to info@indsante.fr. In this context, AD and NPF had access to the data but these legal restrictions prohibit them from making data publicly available.
