# Supplementary material for: Continuous positive airway pressure therapy for obstructive sleep apnoea and psychotropic drug use: a retrospective observational matched-cohort study
Source: Sci Rep. 2018 Sep 20;8:14134. doi: 10.1038/s41598-018-32142-6 (PMC6148046; doi:10.1038/s41598-018-32142-6)
Supplement: Supplementary file 1 — Supplementary Information [file 41598_2018_32142_MOESM1_ESM.pdf]

# Continuous positive airway pressure therapy for obstructive sleep apnoea and psychotropic drug use: a retrospective observational matched-cohort study

Angélique Delbarre[1,*], Frédéric Gagnadoux[2,3], Bénédicte Gohier[4,5], and Nathalie Pelletier-Fleury[1]

[1]Center for research in Epidemiology and Population Health (CESP), Université Paris-Saclay, Université Paris-Sud, UVSQ , Villejuif, France

[2]Université Bretagne Loire, CHU d'Angers, Département de Pneumologie, Angers, France

[3]Inserm UMR 1063 SOPAM, Angers, France

[4]Université Bretagne Loire, Laboratoire de Psychologie des Pays de Loire EA 4638, Angers, France

[5]CHU d'Angers, Service de Psychiatrie et Addictologie, Angers, France

[*]angelique.delbarre@inserm.fr

According to the Classification Commune des Actes Médicaux (CCAM), the classification of medical procedures, sleep recordings (polygraphies or polysomnographies) used to diagnose obstructive sleep apnoea (OSA) were identified with the codes GLQP007, MQP010, AMQP011, AMQP012, AMQP013, AMQP014, AMQP015. Using the Liste des Produits et Prestations Remboursables (LPPR), the classification of medical devices, continuous positive airway pressure (CPAP) were identified with the codes 1100040, 1100637, 1103156, 1103854, 1104405, 1108544, 1113545, 1113930, 1114527, 1115863, 1118614, 1118904, 1119542, 1120723, 1121131, 1122024, 1122337, 1124460, 1124796, 1126275,1126660, 1127688, 1130897, 1133163, 1137221, 1141197, 1141205, 1142541, 1148064, 1149365, 1157459, 1159234, 1162093, 1162124, 1166688, 1172513, 1174742, 1176340, 1182598, 1185361, 1185421, 1188661, 1188684, 1188767, 1189991, 1191864, 1192148, 1196962, 1197128 and mandibular advancement devices (MAD) with the codes 2407378, 2412971, 2451474, 2455325, 2462680, 2497884.

**Supplementary Method S1. Codes from classifications of medical procedures and medical devices.**

| | Database | Type of code | Code | Condition(s) |
|---|---|---|---|---|
| **INCLUSION CRITERIA**[1] | | | | |
| Antidepressants | EGB | ATC | N06A, N05AN01, N03AG01, N03AG02 | Having at least 3 prescriptions of medications at different days during year N |
| Anxiolytics | EGB | ATC | N05BA01, N05BA02, N05BA03, N05BA04, N05BA05, N05BA06, N05BA07, N05BA08, N05BA09, N05BA10, N05BA11, N05BA12, N05BA13, N05BA14, N05BA15, N05BA16, N05BA17, N05BA18, N05BA19, N05BA20, N05BA21, N05BA22, N05BA23, N05BB01, N05BB02, N05BC01, N05BE01, N05BX03 | Having at least 3 prescriptions of medications at different days during year N |
| Hypnotics | EGB | ATC | N05CD02, N05CD03, N05CD04, N05CD05, N05CD06, N05CD07, N05CD08, N05CD09, N05CD10, N05CD11, N05CF01, N05CF02, N05BC51, N05CM11, N05CM16, N05CX | Having at least 3 prescriptions of medications at different days during year N |
| **EXCLUSION CRITERIA**[1] | | | | |
| Neuroleptics | EGB | ATC | N05A (except N05AN01) | Having at least 3 prescriptions of medications at different days during year N |
| Psychotic disorders | EGB, PMSI-MCO | ICD 10 | F20, F21, F22, F23, F24, F25, F28, F29 | Having at least one of the following psychotic disease during year N, N-1, N-2, N-3 or N-4 |
| Mood neurotic disorders | EGB, PMSI-MCO | ICD 10 | F30, F31, F32, F33, F34, F38, F39, F40, F41, F42, F43, F445, F48 | Having at least one of the following psychotic disease during year N, N-1, N-2, N-3 or N-4 |
| Mental disabilities | EGB, PMSI-MCO | ICD 10 | F70, F71, F72, F73, F78, F79 | Having at least one of the following psychotic disease during year N or N-1 |
| Addictive disorders | EGB, PMSI-MCO | ICD 10 | F10, F11, F12, F13, F14, F15, F16, F17, F18, F19 | Having at least one of the following psychotic disease during year N or N-1 |
| Psychiatric disorders from childhood | EGB, PMSI-MCO | ICD 10 | F80, F81, F82, F83, F84, F88, F89, F90, F91, F92, F93, F94, F95, F98 | Having at least one of the following psychotic disease during year N or N-1 |
| Other psychiatric disorders | EGB, PMSI-MCO | ICD 10 | F04, F05 (except F05.1), F06, F07, F09, F50, F51, F52, F53, F54, F55, F59, F60, F61, F62, F63, F64, F65, F66, F68, F69, F99 | Having at least one of the following psychotic disease during year N or N-1 |

**Supplementary Table S1. Algorithm to select the study population at year N.**

EGB: échantillon généraliste des bénéficiaires , PMSI-MCO: programme de médicalisation des systèmes d'information en médecine, chirurgie, obstétrique, ATC: anatomical therapeutic chemical classification system, ICD-10: international classification of diseases, 10th revision.

| **List of World Health Organization**[2] | | | | |
|---|---|---|---|---|
| ATC class | ATC name | Quantity of PAS for one DDD | | |
| N02BE01 | Paracematol | 3 G | | |
| The paracetamol has a DDD of 3 G, which means that an average patient who takes paracetamol for its main indication uses 3 G per day. This is equivalent to 6 standard tablets of 500 MG each. | | | | |
| **Information of a prescription from health insurance database**[3] | | | | |
| Name of drug | ATC class | Number of boxes | Number of tablets in one box | Quantity of PAS in one tablet |
| Doliprane | N02BE01 | 3 | 16 | 500 MG |
| **Number of DDD in a prescription :** | | | | |
| ((Quantity of PAS in one tablet) x (Number of tablets in one box) x (Number of boxes))/Quantity of PAS for one DDD | | | | |
| **There are 8 DDD in this prescription :** | | | | |
| ((500 MG) * (16) * (3))/3 G= 8 DDD | | | | |
| This prescription comprises 8 DDD. | | | | |

**Supplementary Table S2. Example of number of defined daily doses for a prescription.**

WHO: World Health Organisation, ATC: anatomical therapeutic chemical classification system, PAS: pharmaceutical active substance, DDD: defined daily doses, (M)G: (milli)gram(s).

| | OSA individuals group | | | Matched control group | | | P-values* | |
|---|---|---|---|---|---|---|---|---|
| | | mean (±SD) | | | mean (±SD) | | | |
| | n[§] | GP | Psychiatrist | n[§] | GP | Psychiatrist | GP | Psychiatrist |
| Y0 | 869 | 10.01 (±6.59) | 0.68 (±3.53) | 2,607 | 9.67 (±6.55) | 0.61 (±4.38) | 0,180 | 0,624 |
| Y1 | 869 | 9.42 (±6.88) | 0.57 (±3.15) | 2,607 | 8.82 (±6.69) | 0.60 (±3.66) | 0,021 | 0,851 |
| Y2 | 701 | 9.43 (±7.19) | 0.42 (±1.96) | 2,358 | 8.64 (±6.63) | 0.52 (±3.15) | 0,010 | 0,337 |
| Y3 | 479 | 9.74 (±7.16) | 0.42 (±2.00) | 2,083 | 8.76 (±6.99) | 0.54 (±3.21) | 0,010 | 0,293 |

**Supplementary Table S3. Annual visits to the general practitioner and the psychiatrist during the follow-up period.**

OSA: obstructive sleep apnoea, SD: standard deviation, GP: general practitioner, Yi: year i (i=1, 2, 3). §Number of individuals in year Yi (i = 0, 1, 2, 3) knowing that they have not stopped their psychotropic treatment in year Yi-1 (i-1 = 0, 1, 2). *T-tests were performed.

**Model 1: All type of psychotropic drugs**

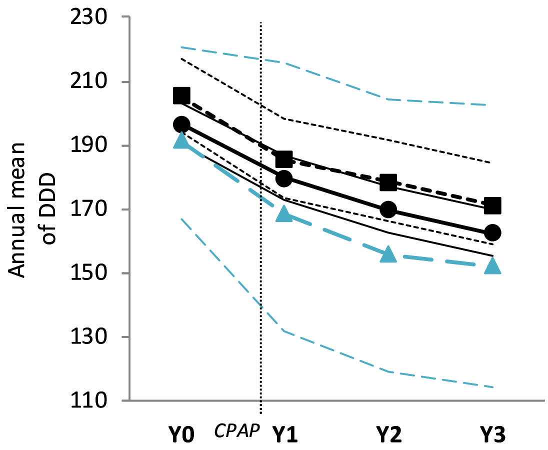


| | Y0 | Y1 | Y2 | Y3 |
|---|---|---|---|---|
| OSA patients group *(n=)* | *869* | *869* | *761* | *546* |
| Non OSA matched control group *(n=)* | *2607* | *2607* | *2537* | *2456* |
| Non adherent OSA individuals *(n=)* | *151* | *151* | *143* | *134* |

**Model 2: Antidepressants**

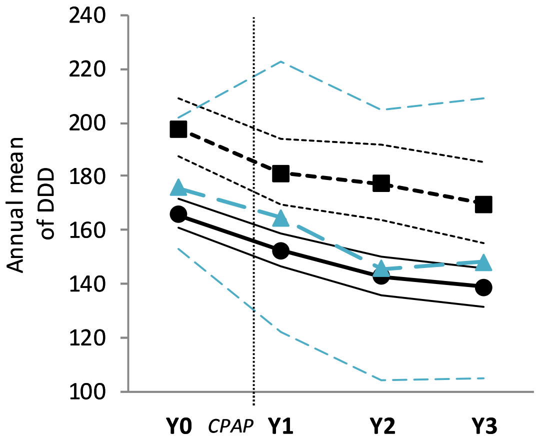


| | Y0 | Y1 | Y2 | Y3 |
|---|---|---|---|---|
| OSA patients group *(n=)* | *540* | *540* | *474* | *329* |
| Non OSA matched control group *(n=)* | *1620* | *1620* | *1584* | *1541* |
| Non adherent OSA individuals *(n=)* | *84* | *84* | *81* | *76* |

**Model 3: Anxiolytics**

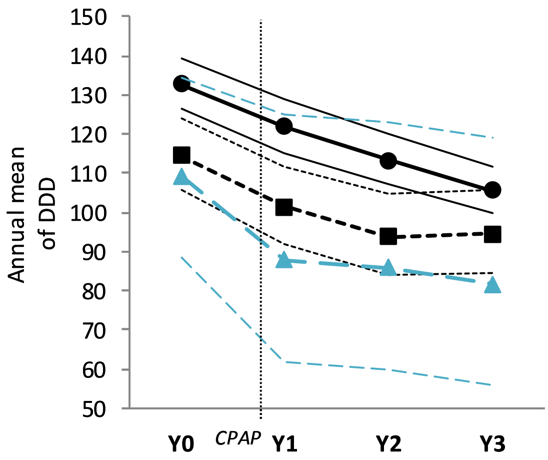


| | Y0 | Y1 | Y2 | Y3 |
|---|---|---|---|---|
| OSA patients group *(n=)* | *392* | *392* | *341* | *235* |
| Non OSA matched control group *(n=)* | *1176* | *1176* | *1140* | *1099* |
| Non adherent OSA individuals *(n=)* | *81* | *81* | *77* | *72* |

**Model 4: Hypnotics**

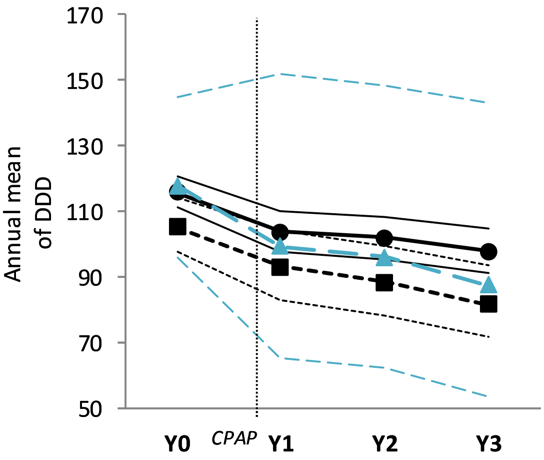


| | Y0 | Y1 | Y2 | Y3 |
|---|---|---|---|---|
| OSA patients group *(n=)* | *253* | *253* | *221* | *172* |
| Non OSA matched control group *(n=)* | *759* | *759* | *730* | *704* |
| Non adherent OSA individuals *(n=)* | *45* | *45* | *41* | *37* |

**Supplementary Figure S1. Results of GEE models on OSA patients and non-OSA matched controls, and results of GEE models on non adherent OSA individuals: estimated annual mean psychotropic drug DDD per group and their 95% CI.**

GEE: generalized estimating equations, DDD: defined daily doses, CI: confidence interval, OSA: obstructive sleep apnoea. Mean and 95% confidence intervals were computed with Generalized Estimating Equations (GEE) models.
